# Association of sorting and assembly machinery component 50 homolog gene polymorphisms with nonalcoholic fatty liver disease susceptibility

**DOI:** 10.1097/MD.0000000000029958

**Published:** 2022-07-22

**Authors:** Ming Qiao, Jian-hua Yang, Yi Zhu, Jun-ping Hu

**Affiliations:** a Department of Pharmacy, The First Affiliated Hospital of Xinjiang Medical University, Urumqi, China; b College of Pharmacy, Xinjiang Medical University, Urumqi, China.

**Keywords:** gene polymorphism, meta-analysis, nonalcoholic fatty liver disease, SAMM50

## Abstract

Sorting and assembly machinery component 50 homolog (SAMM50) gene single-nucleotide polymorphisms (SNPs) have been connected with the susceptibility of nonalcoholic fatty liver disease (NAFLD), but with inconsistent results across the current evidence. The present work was schemed to explore the association between SAMM50 gene SNPs and NAFLD vulnerability via meta-analysis.

**Methods:**

PubMed, Web of Science, Cochrane Library, China National Knowledge Infrastructure (CNKI), and Wanfang were retrieved for eligible literature previous to June 10, 2021. The odds ratios (ORs) of the dichotomic variables and the standardized mean difference of quantitative variables with corresponding 95% confidence intervals (95% CIs) were computed to evaluate the strength of the associations. The quality of included studies was assessed using Newcastle-Ottawa Scale (NOS).

**Results:**

In total, 8 case-control studies encompassing 6297 NAFLD patients and 7306 disease-free controls in this meta-analysis. Ultimately, this analysis included 8, 6, and 5 studies for *rs2143571*, *rs3761472*, and *rs738491* polymorphisms respectively. The pooled data revealed that the 3 polymorphisms had conspicuous associations with NAFLD susceptibility: *rs2143571*, A vs. G, OR=1.51, 95% CI, 1.37–1.66, *P* < .01; *rs3761472*, A vs. G, OR=1.50, 95% CI, 1.35–1.67, *P* < .01; *rs738491*, A vs. G, OR=1.51, 95% CI, 1.40–1.63, *P* < .01.

**Conclusion:**

This meta-analysis suggests that *rs2143571*, *rs3761472*, and *rs738491* polymorphisms of the SAMM50 gene are appreciably associated with augmented risk of NAFLD vulnerability. It will provide the latest evidence to support the susceptibility of SAMM50 gene polymorphisms and NAFLD, and provide strategies for the prevention and treatment of NAFLD.

## 1. Introduction

Nonalcoholic fatty liver disease (NAFLD) is an assessment of fatty infiltration in the liver by histopathologic examination in the absence of excessive alcohol intake, autoimmune, viral infection, or drug-induced liver disease.^[1]^ It is gradually considered the liver disease component of metabolic syndrome, which is a risk factor for further development of fatty liver, nonalcoholic steatohepatitis (NASH), fibrosis, cirrhosis, and hepatocellular carcinoma.^[2–4]^ The global burden of NAFLD is rapidly increasing and is expected to become the most common indication for liver transplantation with the alarming rise in the prevalence of obesity and metabolic syndrome.^[5]^ According to the World Gastroenterology Organization, NAFLD has been increasing during the past 20 years and is now one of the most common types of liver disease in Western countries.^[6]^ According to the present data, NAFLD affects 10%–30% of the general population in various countries, which has been viewed as a huge global health burden.^[7,8]^

Little is known about the latent mechanism involved in the development and pathogenesis of NAFLD, and yet it is a complicated metabolic process in which both environmental and genetic factors are etiology.^[9]^ At present, genome-wide association studies (GWAS) have demonstrated several conspicuous genetic susceptibility genes such as PNPLA3, SAMM50, PARVB, APOC3, PPAR-γ, NCANCILP2, FABP1, AGTR1, GSTs, which had been verified the crucial roles in the disease onset and progression of NAFLD.^[10–15]^ Among them, *rs738491*, *rs2143571*, and *rs3761472* in the sorting and assembly machinery component 50 homolog (SAMM50) gene were demonstrated to be notably associated with NAFLD susceptibility.^[16]^ Up to now, there have been several reports on SAMM50 gene SNPs and NAFLD susceptibility, but the results were inconsistent. Additionally, the real situation of the whole population cannot be reflected due to the limited sample size and regional and ethnic differences in the existing single studies. Therefore, a meta-analysis will be conducted on the existing research results to obtain more authentic and reliable results.

## 2. Materials and Methods

### 2.1. Literature collection

This study depended on previously published literature and public databases. Thus, this work does not need ethical approval and patient consent. Two independent researchers searched the PubMed, Web of Science, Cochrane Library, CNKI, and Wanfang databases before June 10, 2021. The searching strategy of PubMed was exhibited as follows: (“Nonalcoholic fatty liver disease [Mesh]” OR Non-alcoholic fatty liver disease OR NAFLD OR Non-alcoholic steatohepatitis OR NASH) AND (“Sorting and assembly machinery component 50 homolog” [Mesh] OR SAMM50) AND (“Single Nucleotide Polymorphism” [Mesh] OR Variant OR SNP OR Polymorphism OR Mutant OR Mutation OR Variation). No language restriction was set. This study depended on previously published literature and public databases. Thus, this work does not need ethical approval and patient consent.

### 2.2. Inclusion and exclusion criteria

Original studies were incorporated into this analysis in the light of the following inclusion criteria: (i) case-control studies; (ii) study investigated the associations of SAMM50 polymorphisms and NAFLD predisposition; (iii) NAFLD is diagnosed by pathology or ultrasound; (iv) control subjects were disease-free individuals; (v) detailed genotype data can be calculated for ORs and 95% CIs. Correspondingly, the exclusion criteria were as follows: reviews, conference abstracts, commentary articles, letters to the editor, animal studies, unpublished data, case reports, and secondary causes of steatosis, including alcoholism, total parenteral nutrition, hepatitis B/C virus infection, and drug usage.

### 2.3. Methodological quality assessment

The Newcastle–Ottawa Scale (NOS) was performed to evaluate the methodological quality of included studies by 2 independent investigators. The NOS is composed of 3 aspects, namely selection, comparability, and exposure. Each study could receive 0 to 9 scores. Nevertheless, studies with ≥6 scores were regarded as high-quality studies. Disagreement was resolved by discussion.

### 2.4. Data extraction

The extracted information contained the name of the first author, year of publication, country, ethnicity, numbers of NAFLD and control subjects, diagnostic methods of NAFLD, genotype frequencies, and Hardy–Weinberg equilibrium (HWE) results. Two investigators extracted the information independently.

### 2.5. Statistical analysis

In the case of polymorphism with 2 alleles (A1 and A2), both the NAFLD and disease-free participants could be classified into 4 exposure groups (A1, A1A1, A1A2, or A2A2). Because the underlying genetic model for SAMM50 gene polymorphisms and NAFLD was unclear, the estimation of the association was based on 5 genetic models including allele contrast (A1 vs. A2), homozygous contrast (A1A1 vs. A2A2), heterozygous contrast (A1A2 vs. A2A2), dominant contrast (A1A1 + A1A2 vs. A2A2), and recessive contrast (A1A1 vs. A1A2 + A2A2). The control participants of incorporated studies were estimated via HWE.^[17]^ Summary ORs with 95% CIs were computed to specifically evaluate the relationship between SAMM50 gene polymorphisms and NAFLD susceptibility. Cochran’s *Q*-test and *I*^2^ test were applied to appraise the between-study heterogeneity.^[18]^ The random-effect model was employed to calculate merged ORs if *P* < .1, *I*^2^ > 50%. If not, the fixed-effect model was utilized for data synthesis.^[19]^ Sensitivity analysis was performed as well to assess the stability of all incorporated studies via the leave-one-out method. When the included studies were more than 10, a funnel plot was employed to evaluate the publication bias.^[20]^ On the contrary, less than 10 studies are not required. All analyses were done using Review Manager 5.3 software (Nordic Cochrane Centre, Cochrane Collaboration, Copenhagen, Sweden).

## 3. Results

### 3.1. Literature search

**Figure 1.**
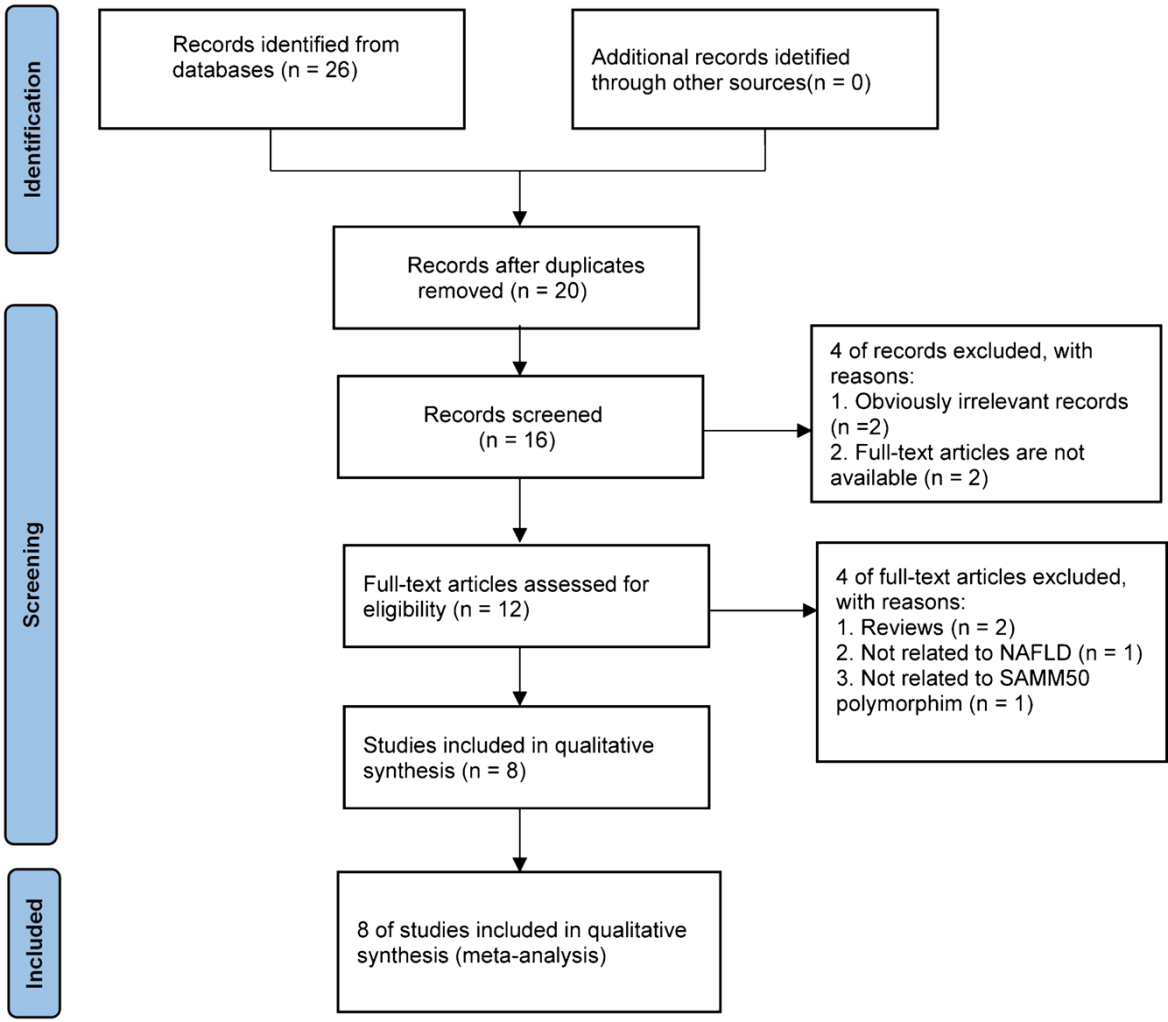
Flowchart of literature search and screen.

A primary search of electronic databases retrieved 26 potentially relevant publications: 13 from PubMed, 12 from Web of Science, 0 from Cochrane Library, 0 from CNKI, and 1 from Wanfang. No additional records were acquired from other sources. And then 20 studies remained after removing duplicated articles. Subsequently, a total of 4 irrelevant articles were excluded based on titles and abstracts screening. After applying inclusion and exclusion criteria, 3 unrelated articles (not related to NAFLD/SAMM50 gene polymorphisms), and 3 reviews were removed and 2 full-text article was not available. Ultimately, 8 studies (Kawaguchi et al., 2012; Kitamoto et al., 2013; Kitamoto et al., 2014; Kanth et al., 2014; Chen et al., 2015; Edelman et al., 2015; Larrieta et al., 2015; Chung et al., 2018) went into the process of meta-analysis. Overall, a flowchart summarizing the procedure of literature identification was illustrated in Figure 1.

### 3.2. Main characteristics

The studies were performed in Japan,^[21–23]^ India,^[24]^ China,^[25]^ USA,^[26]^ Mexico^[27]^, and Korea.^[28]^ They were all case-control designed and published between 2012 and 2018. It is worth noting that 2 studies (Kitamoto et al., 2013; Chung et al., 2018) were genome-wide association studies (GWAS), including 2 stages of discovery I and validation II. In total, 8 studies encompassing 6297 cases and 7306 control participants were analyzed in the current analysis. For rs2143571, rs3761472, and rs738491 polymorphisms, there were 8, 6, and 5 studies ultimately incorporated, respectively. The main characteristics of the included literature were shown in Table 1. It should be noted that the study of Chung et al.^[28]^ and Kanth et al^[24]^ did not provide the genotype frequency of the control group, so HWE could not be calculated. Studies with≥6 scores were regarded as high-quality studies according to the evaluation of methodological quality (Table 2).

**Table 1:** Main characteristics of the included studies.

						Case				Control				*P*_HWE_
Study	Year	Country	Ethnicity	Means of diagnosis	Sample size (case/control)	A1	A1A1	A1A2	A2A2	A1	A1A1	A1A2	A2A2	
***Rs2143571***														
Kawaguchi	2012	Japan	Asian	Liver biopsy	529/932	587	167	253	109	854	194	466	272	.83
Kitamoto I	2013	Japan	Asian	Liver biopsy	390/934	433	124	185	81	795	184	427	323	.53
Kitamoto II	2013	Japan	Asian	Liver biopsy	172/1012	192	53	86	33	860	171	518	323	.13
Kitamoto	2014	Japan	Asian	Liver biopsy	540/1012	292	NA	NA	NA	425	NA	NA	NA	NA
Kanth	2014	India	Asian	Liver biopsy	156/150	97	NA	NA	NA	60	NA	NA	NA	NA
Chen	2015	China	Asian	Ultrasound	340/452	376	112	152	76	366	76	214	162	.71
Edelman	2015	USA	Caucasian	Liver biopsy	39/24	34	9	16	14	22	1	10	13	.59
Larrieta	2018	Mexico	Caucasian	Ultrasound	178/453	154	33	88	57	304	48	208	197	.53
Chung I	2018	Korea	Asian	Ultrasound	2816/1593	2551	NA	NA	NA	1258	NA	NA	NA	NA
Chung II	2018	Korea	Asian	Ultrasound	1137/744	1018	NA	NA	NA	565	NA	NA	NA	NA
***Rs3761472***														
Kawaguchi	2012	Japan	Asian	Liver biopsy	528/932	582	162	258	108	851	195	461	276	.92
Kitamoto I	2013	Japan	Asian	Liver biopsy	392/934	438	126	186	80	786	178	430	326	.09
Kitamoto II	2013	Japan	Asian	Liver biopsy	172/1011	192	53	86	33	851	168	515	328	.12
Kitamoto	2014	Japan	Asian	Liver biopsy	540/1012	292	NA	NA	NA	425	NA	NA	NA	NA
Chen	2015	China	Asian	Ultrasound	340/452	357	102	153	85	357	72	213	167	.77
Edelman	2015	USA	Caucasian	Liver biopsy	39/24	30	8	14	17	8	0	8	16	.33
Chung I	2018	Korea	Asian	Ultrasound	2816/1593	2534	NA	NA	NA	1243	NA	NA	NA	NA
Chung II	2018	Korea	Asian	Ultrasound	1137/744	1026	NA	NA	NA	562	NA	NA	NA	NA
***Rs738491***														
Kawaguchi	2012	Japan	Asian	Liver biopsy	529/932	667	214	239	76	1010	271	468	193	.73
Kitamoto I	2013	Japan	Asian	Liver biopsy	392/934	494	162	170	60	980	266	448	220	.24
Kitamoto II	2013	Japan	Asian	Liver biopsy	172/1011	219	66	87	19	1000	247	506	258	.97
Kitamoto	2014	Japan	Asian	Liver biopsy	540/1012	335	NA	NA	NA	496	NA	NA	NA	NA
Chen	2015	China	Asian	Ultrasound	340/452	397	123	151	66	414	94	226	132	.88
Larrieta	2018	Mexico	Caucasian	Ultrasound	177/451	161	38	85	54	338	69	200	182	.25

A1/A2 = allelic gene; HWE = Hardy–Weinberg equilibrium; NA = not reported.

**Table 2:** Quality assessment of included studies based upon the Newcastle–Ottawa Scale (NOS).

Item/Study	Kawaguchi2012	Kitamoto 2013	Kitamoto 2014	Kanth 2014	Chen 2015	Edelman 2015	Larrieta 2018	Chung 2018
Selection								
Adequate definition of cases	1	1	1	1	1	1	1	1
Representativeness of cases	1	1	1	1	0	0	0	1
Selection of control subjects	1	0	0	0	0	1	0	1
Definition of control subjects	1	1	1	1	1	1	1	1
Comparability								
Control for important factor or additional factor	1	1	1	1	1	1	1	1
Exposure								
Exposure assessment	1	1	1	1	1	0	1	1
Same method of ascertainment for all subjects	1	1	1	1	1	1	1	1
Non-response rate	1	1	1	1	1	1	1	1
Total score	8	7	7	7	6	6	6	8

### 3.3. Rs2143571 polymorphism and NAFLD susceptibility

**Figure 2.**
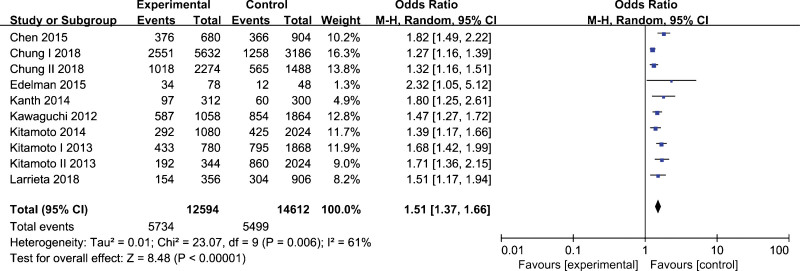
Forest plot of the association between *rs2143571* polymorphism and nonalcoholic fatty liver disease under allelic model (A vs. G).

In a total of 8 studies (Kawaguchi et al., 2012; Kitamoto et al., 2013; Kitamoto et al., 2014; Kanth et al., 2014; Chen et al., 2015; Edelman et al., 2015; Larrieta et al., 2015; Chung et al., 2018) including 6297 NAFLD patients and 7306 controls for *rs2143571* polymorphism. The allelic model was analyzed using the random-effect model due to significant heterogeneity. No remarkable heterogeneity was found in other genetic models, and a fixed-effect model was employed for data analysis. The results indicated that *rs2143571* polymorphism was significantly associated with NAFLD susceptibility under the allelic model (A vs. G, OR=1.51, 95% CI, 1.37–1.66, *P* < .01), homozygous model (AA vs. GG, OR=2.61, 95% CI, 2.20–3.10, *P* < .01), heterozygous model (AG vs. GG, OR=1.51, 95% CI, 1.31–1.75, *P* < .01), dominant model (AA+AG vs. GG, OR=1.81, 95% CI, 1.58–2.08, *P* < .01), and recessive model (AA vs. AG+GG, OR=1.99, 95% CI, 1.73–2.29, *P* < .01) (Table 3). Carriers of the A-allele have a higher risk of NAFLD, as shown in Figure 2.

**Table 3:** Association between SAMM50 gene polymorphisms and NAFLD susceptibility.

	Test of association				Test of heterogeneity	
Genetic model	OR	95% CI	*P* value	Statistical model	*I*^*2*^(%)	*P* value
*Rs2143571*						
A vs. G	1.51	1.37–1.66	<.01	R	61	<.01
AA vs. GG	2.61	2.20–3.10	<.01	F	0	.55
AG vs. GG	1.51	1.31–1.75	<.01	F	0	.88
AA+AG vs. GG	1.81	1.58–2.08	<.01	F	0	.80
AA vs. AG+GG	1.99	1.73–2.29	<.01	F	0	.53
*Rs3761472*						
A vs. G	1.50	1.35–1.67	<.01	R	66	<.01
AA vs. GG	2.64	2.20–3.16	<.01	F	6	.37
AG vs. GG	1.55	1.32–1.81	<.01	F	0	.83
AA+AG vs. GG	1.85	1.60–2.15	<.01	F	0	.68
AA vs. AG+GG	1.97	1.71–2.28	<.01	F	11	.34
*Rs378491*						
A vs. G	1.51	1.40–1.63	<.01	F	0	.45
AA vs. GG	2.31	1.94–2.76	<.01	F	13	.33
AG vs. GG	1.44	1.23–1.70	<.01	F	0	.41
AA+AG vs. GG	1.73	1.48–2.02	<.01	F	7	.37
AA vs. AG+GG	1.79	1.57–2.03	<.01	F	0	.64

### 3.4. Rs3761472 polymorphism and NAFLD susceptibility

**Figure 3.**
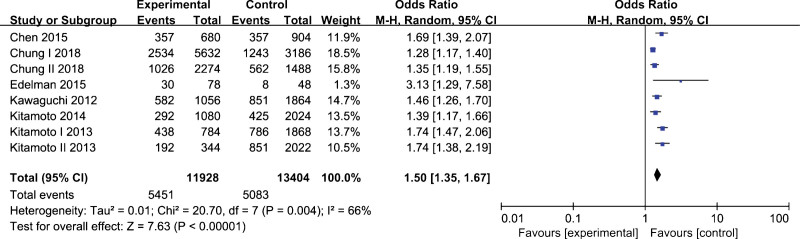
Forest plot of the association between *rs3761472* polymorphism and nonalcoholic fatty liver disease under allelic model (A vs. G).

Overall, 6 research containing 5964 NAFLD and 6702 healthy subjects for *rs3761472* to perform data analysis. The allelic model was analyzed by a random-effect model owing to significant heterogeneity observed. As the heterogeneity of genetic models was not significant, the fixed-effect model was employed for data analysis. The pooled data indicated there was a noticeable association between *rs3761472* polymorphism and NAFLD susceptibility under allelic model (A vs. G, OR=1.50, 95% CI, 1.35–1.67, *P* < .01), homozygous model (AA vs. GG, OR=2.64, 95% CI, 2.20–3.16, *P* < 0.01), heterozygous model (AG vs. GG, OR=1.55, 95% CI 1.32–1.81, *P* < 0.01), dominant model (AA+AG vs. GG, OR=1.85, 95% CI, 1.60–2.15, *P* < .01), and recessive model (AA vs. AG+GG, OR=1.97, 95% CI, 1.71–2.28, *P* < .01) (Table 3). Carriers of the A-allele have a higher risk of NAFLD, as shown in Figure 3.

### 3.5. Rs738491 polymorphism and NAFLD susceptibility

**Figure 4.**
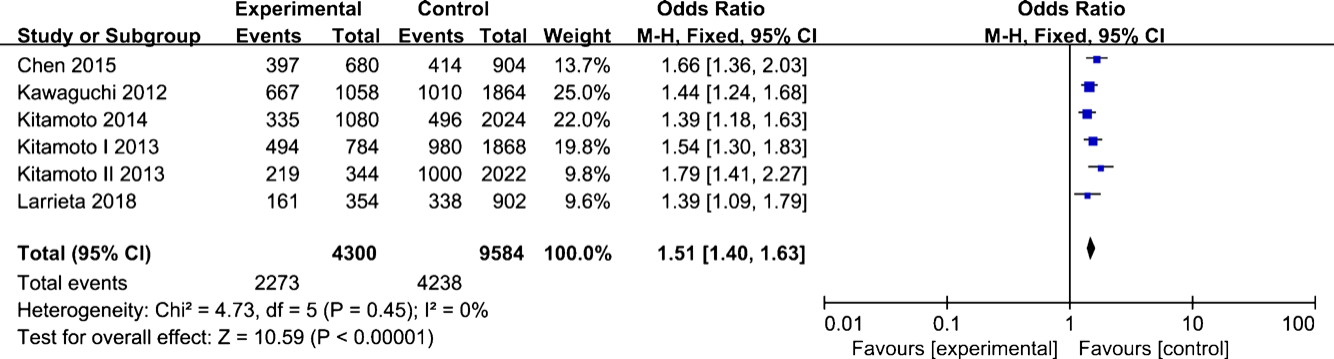
Forest plot of the association between *rs738491* polymorphism and nonalcoholic fatty liver disease under allelic model (A vs. G).

A total of 5 studies with 2150 cases and 4792 controls were used for data pooled. The fixed-effect model was employed for data analysis owing of the between-study heterogeneity was not significant. The results indicated an obvious association between *rs738491* polymorphism and NAFLD susceptibility under allelic model (A vs. G, OR=1.51, 95% CI, 1.40–1.63, *P* < .01), homozygous model (AA vs. GG, OR=2.31, 95% CI, 1.94–2.76, *P* < .01), heterozygous model (AG vs. GG, OR=1.44, 95% CI, 1.23–1.70, *P* < .01), dominant model (AA+AG vs. GG, OR=1.73, 95% CI, 1.48–2.02, *P* < .01), and recessive model (AA vs. AG+GG, OR=1.79, 95% CI 1.57–2.03, *P* < .01) (Table 3). Carriers of the A-allele have a higher risk of NAFLD, as shown in Figure 4.

### 3.6. Sensitivity analysis and publication bias

After the omission of an individual study, the recalculated *P* value, ORs, and 95% CIs did not change substantially. Therefore, the outcomes were considered to be statistically robust and reliable. The funnel plot for assessment of publication bias was not implemented on account of fewer than 10 research.

## 4. Discussion

Despite the specific pathological mechanism of NAFLD still needs to be explored. Nevertheless, research increasingly revealed that genetic predisposition plays a crucial intrinsic role in the occurrence and development of NAFLD.^[29]^ In addition, SNPs in humans might be one of the critical steps to disclosing the genetic factor for NAFLD pathogenesis. In recent years, researchers have found that many gene polymorphisms are associated with NAFLD vulnerability through GWAS. Kawaguchi et al.^[21]^ were the first to find a significant association between SAMM50 gene polymorphisms and NAFLD susceptibility in the Japanese population via GWAS. Subsequently, the study was carried out by researchers from different countries, but the results were inconsistent. This inconsistency might be caused by factors like limited sample sizes, confounding factors, as well as clinical heterogeneity of NAFLD. Therefore, we collected the existing evidence and looked into the associations of SAMM50 gene SNPs and NAFLD vulnerability via meta-analysis, which could combine data from individual studies, examine and explain the heterogeneity, and increase the statistical power. In conclusion, the merged data suggested a significant correlation between *rs2143571*, *rs3761472*, and *rs738491* polymorphisms of the SAMM50 gene and NAFLD vulnerability. Two large samples of GWAS were included in the original studies, with sufficient assurance to ensure the reliability of the study results. Of note, rigid methodological quality assessment, heterogeneity, and sensitivity analysis also ensured the robustness of the study results.

In a word, we performed a meta-analysis of 8 case-control studies that satisfied the inclusion criteria. It should be noted that the current comprehensive analysis was more necessary and meaningful owing to the conclusions of qualified case-control studies are conflicting and contradictory. This work demonstrated that carriers of the A-allele of *rs2143571*, *rs3761472*, and *rs738491* in the SAMM50 gene have a higher risk of NAFLD. Namely, it has a significantly increased risk for NAFLD. Furthermore, the outcomes of the meta-analysis were considered to be statistically robust and reliable according to the sensitivity analysis.

Sam50 is a kind of β-barrel protein distributed in the mitochondrial membrane and encoded by the SAMM50 gene.^[30]^ It plays an important role in the formation of the mitochondrial crest, the assembly of the respiratory chain complex, and the maintenance of mitochondrial DNA stability.^[31]^ Studies have revealed that Sam50 reduction may lead to changes in mitochondrial shape and cristal morphology, and SAM50 consumption could affect the mitochondrial respiratory complexes.^[32]^ Ma et al.^[33]^ found a significant deletion of mitochondrial cristae in liver cells of patients with nonalcoholic steatohepatitis. In addition, changes in the normal structure of mitochondria could lead to insulin resistance, which is an important pathophysiological process of NAFLD.^[34,35]^ All the above studies have indicated that the SAMM50 gene and its expression products play a crucial role in the development of NAFLD. According to the results of this meta-analysis, A allele of *rs2143571*, *rs3761472*, and *rs738491* in the SAMM50 gene might be related to mitochondrial dysfunction and insulin resistance, leading to the NAFLD development.

Up to now, this is the first synthetical study on the relationship between SAMM50 gene SNPs and NAFLD vulnerability. There were several strengths in this study. First, to gather a maximum amount of relevant literature, a comprehensive search strategy was adopted to retrieve eligible studies in both English and Chinese databases. Besides, the methodological quality of studies was evaluated via NOS, which allowed for the judgment of the potential risk of bias. According to the NOS, all eligible studies were of high methodological quality. Furthermore, sensitivity analyses were carried out in this study, which guaranteed the reliability of the findings.

Nevertheless, there still existed several drawbacks that should be acknowledged. First, Although liver biopsy was regarded as the gold standard for NAFLD diagnosis, not all studies were included liver biopsy because pathology was expensive and invasive. Second, the original studies included in this study were mainly conducted in Asian and North American populations, and the results cannot reflect the situation of other populations. Third, We ignored the synergistic effect of polymorphisms at other sites of NAFLD because only 3 loci in the SAMM50 gene were studied in association with susceptibility to NAFLD. Thus, interactions between these loci and genes may result in concealing or amplifying the actual function of individual loci or genes. Leave aside these drawbacks, this study is the first to provide more accurate and powerful evidence on the association between *rs2143571*, *rs3761472*, *and rs738491* polymorphisms of the SAMM50 gene and NAFLD vulnerability.

## 5. Conclusion

This meta-analysis suggests that *rs2143571*, *rs3761472*, and *rs738491* polymorphisms of the SAMM50 gene are appreciably associated with an augmented risk of NAFLD vulnerability. In summary, this study will provide the latest evidence to support the susceptibility of SAMM50 gene polymorphisms and NAFLD, and provide strategies for the prevention and treatment of NAFLD. Concerning the limitations of this study, it is necessary to confirm the present findings with complementary studies with a larger sample size.

## Acknowledgments

The authors would like to thank all studies and their participants involved in this investigation.

## Author contributions

Conceptualization: Ming Qiao.

Supervision: Jian-hua Yang, Jun-ping Hu.

Validation: Yi Zhu.

Writing–original draft: Ming Qiao.

Writing–review & editing: Jian-hua Yang, Jun-ping Hu.
